# Diarrhea and dengue control in rural primary schools in Colombia: study protocol for a randomized controlled trial

**DOI:** 10.1186/1745-6215-13-182

**Published:** 2012-10-03

**Authors:** Hans J Overgaard, Neal Alexander, Maria Ines Mátiz, Juan Felipe Jaramillo, Victor Alberto Olano, Sandra Vargas, Diana Sarmiento, Audrey Lenhart, Razak Seidu, Thor Axel Stenström

**Affiliations:** 1 Department of Mathematical Sciences and Technology, Norwegian University of Life Sciences, Ås, Norway; 2 London School of Hygiene and Tropical Medicine, London, UK; 3 Instituto de Salud y Ambiente, Universidad El Bosque, Bogotá, Colombia; 4 Liverpool School of Tropical Medicine, Liverpool, UK

**Keywords:** Dengue, Diarrhea, Primary schools, Rural, Children, Mosquito, Water, Prevention

## Abstract

**Background:**

Diarrheal diseases and dengue fever are major global health problems. Where provision of clean water is inadequate, water storage is crucial. Fecal contamination of stored water is a common source of diarrheal illness, but stored water also provides breeding sites for dengue vector mosquitoes. Poor household water management and sanitation are therefore potential determinants of both diseases. Little is known of the role of stored water for the combined risk of diarrhea and dengue, yet a joint role would be important for developing integrated control and management efforts. Even less is known of the effect of integrating control of these diseases in school settings. The objective of this trial was to investigate whether interventions against diarrhea and dengue will significantly reduce diarrheal disease and dengue entomological risk factors in rural primary schools.

**Methods/design:**

This is a 2×2 factorial cluster randomized controlled trial. Eligible schools were rural primary schools in La Mesa and Anapoima municipalities, Cundinamarca, Colombia. Eligible pupils were school children in grades 0 to 5. Schools were randomized to one of four study arms: diarrhea interventions (DIA); dengue interventions (DEN); combined diarrhea and dengue interventions (DIADEN); and control (C). Schools were allocated publicly in each municipality (strata) at the start of the trial, obviating the need for allocation concealment. The primary outcome for diarrhea is incidence rate of diarrhea in school children and for dengue it is density of adult female *Aedes aegypti* per school. Approximately 800 pupils from 34 schools were enrolled in the trial with eight schools in the DIA arm, nine in the DEN, eight in the DIADEN, and nine in the control arms. The trial status as of June 2012 was: completed baseline data collections; enrollment, randomization, and allocation of schools. The trial was funded by the Research Council of Norway and the Lazos de Calandaima Foundation.

**Discussion:**

This is the first trial investigating the effect of a set of integrated interventions to control both dengue and diarrhea. This is also the first trial to study the combination of diarrhea-dengue disease control in school settings.

**Trial registration:**

Current Controlled Trials ISRCTN40195031

## Background

Diarrheal diseases and dengue fever are major global health problems. In areas without a regular, safe water supply, water is frequently stored in containers in and around homes, providing abundant, ideal habitats for dengue vectors to breed. Fecal contamination of stored water used for ingestion is also a source of diarrheal illness. Containers used to intentionally store water may thus be a common denominator for both diseases. Effective control of both diarrheal diseases and dengue depends on the provision of a reliable supply of safe water, appropriate water management practices, and community participation in control efforts [1,2]. Integrated interventions that target both diseases are likely to be both effective and cost-efficient.

Globally, about 88% of the diarrheal disease burden is due to unsafe water supply and lack of sufficient sanitation and hygiene [1,3]. An estimated 780 million people lack access to safe water sources and 2.5 billion do not use improved sanitation [4]. Despite these high numbers, substantial improvements have been achieved during the last decades evidenced by 89% of the global population currently using improved drinking water sources and 63% having access to improved sanitation. In fact the target of the Millennium Development Goal to reduce by half the proportion of people without sustainable access to safe drinking water has been met [4]. However, about 4 billion cases of diarrhea still occur annually, killing about 2 million people, primarily children in developing countries [1,5]. Infections usually arise through ingestion of water contaminated with human or animal feces [6]. Inadequate domestic water supply, for example absent or irregular piped water, forces people to collect water and store it in or near houses. Microbial contamination between source and point-of-use is often a significant cause of reduced water quality [7].

Dengue fever is the most rapidly spreading vector-borne disease globally. Around 50 million cases, mainly children, occur annually in about 100 countries [8] and about 2.5 billion people live in risk areas [9]. Dengue, and its more severe manifestation, dengue hemorrhagic fever (DHF), are caused by a flavivirus with four different serotypes. Dengue is transmitted primarily by the *Aedes aegypti* mosquito, which preferentially breeds in artificial water containers in close proximity to human habitation. Dengue transmission risk increases with rapid, unplanned, and unregulated urban development, poor water storage, and unsatisfactory sanitary conditions [8,10-13]. As no effective dengue vaccine or therapeutic drugs are available, vector control is the only way to prevent dengue transmission.

Diarrhea and dengue are both highly endemic throughout Latin America and the Caribbean. In 2000, an estimated 71.5 million people lacked access to safe drinking water in this region [14]. In 2000, 76% of Colombian municipalities did not have potable water, and an estimated 60% of the inhabitants in rural areas had a medium to high risk of contracting diseases because of poor water quality [15]. In 2009, 14.4 million people were at high risk of water shortage and only 40% of households had both water connection and sewage [16]. In 20% of Colombian municipalities (222 of 1,102) the water supply coverage in rural areas was <30% and in as many as 54% of all Colombian municipalities the sewage coverage in rural areas was <30% [16]. Region-wide infant mortality rates from diarrheal disease were 3.7% during 2000 to 2005, but in the Andean sub-region, including Colombia, the rate was 7.8% [17]. Diarrhea is a leading cause of morbidity and one of the 10 most important in terms of mortality in Colombia [18], with an estimated prevalence of 13% in children <5 years old [19]. In 2008, the Pan-American Health Organization (PAHO) reported that Brazil, Venezuela, México, and Colombia had the highest number of dengue cases in the Americas. In Colombia, about 65% of the urban population is considered to be at high risk of contracting dengue. Dengue hemorrhagic fever (DHF) incidence in Colombia constitutes 58.6% of all DHF in the Andean region, and 30% of all DHF in the Americas [15]. All four dengue virus serotypes circulate in Colombia and in addition to *Ae. aegypti*, a secondary dengue vector, *Aedes albopictus*, has become established in western Colombia [20,21]. In Colombia, as in many other countries, the organophosphate larvicide temephos is commonly added to water storage containers as a key component of dengue vector control programs. Although temephos is safe for human consumption [22], temephos treatment often encounters strong opposition from householders when the water is used for drinking, as it can cause the water to appear cloudy and has a disagreeable taste. Lack of a chemical barrier to mosquito breeding puts drinking water containers at a potentially higher risk of becoming dengue vector breeding sites. Resistance to temephos in *Aedes aegypti* has been identified in many locations in Colombia, including in the current study area [23-25].

Studies from the Caribbean region indicate that poor provision of reliable drinking water supply and waste disposal services was largely responsible for *Ae. aegypti* propagation [26,27]. In Colombia, householders often keep a stored supply of water in the home, even in areas with access to piped water. On this country’s Caribbean coast, household water storage tanks and drums were found responsible for producing up to 95% of *Aedes aegypti* pupae [28]. These same containers were also shown to be the primary dengue vector breeding sites in studies in Antioquia and Cundinamarca provinces in central Colombia [29,30]. While the water stored in these containers is often used for washing and cooking, it can also be used for human consumption. Although little published research is available on the epidemiology of diarrheal illness in Colombia, lack of access to reliable, clean drinking water is likely a key factor in making it a leading cause of morbidity, particularly among children.

There is little existing information on the functional relationships between diarrhea and dengue fever. A literature search reveals few studies where risk factors of the two diseases have been studied simultaneously and how one affects the other. Full-text searches of the Cochrane database, Web of Science^SM^, and Pub Med using the search terms ‘dengue’, ‘dengue fever’, ‘diarrhea’, and ‘diarrhoea’ and combinations thereof yielded no relevant studies. As no studies of this kind have been carried out in schools, little is known of how stored water influences the risk of diarrhea and dengue and how interventions against both diseases affect children in school settings.

Our study focuses on schools for two key reasons. First, the morning biting peak of the local dengue vector occurs when children are likely to be in school [30]. If schools are important dengue vector breeding grounds, children attending school may be disproportionately exposed. Second, only about 37% of the schools in the study area have access to potable water [31], potentially exposing pupils to diarrheal pathogens from water ingested at school.

This study protocol description follows the CONSORT statement extension to cluster randomized trials [32].

### Objectives

This trial will investigate whether a set of disease-specific interventions will significantly reduce diarrheal cases and dengue entomological risk factors in rural primary schools in two municipalities in Colombia. The hypothesis is that the interventions will significantly reduce the number of diarrheal disease cases, the number of school absence episodes, dengue vector infestation, and water contamination as compared to schools that do not receive the interventions.

Specifically, we hypothesize that the interventions will:

(1) Reduce exposure to diarrhea risk factors in schools by:

a. improving drinking water quality;

b. improving hand-washing practices;

c. improving sanitary hygiene;

d. improving health education on diarrheal disease prevention.

(2) Reduce exposure to dengue risk factors in schools by:

a. reducing mosquito entry to school classrooms by insecticide-treated curtains;

b. reducing dengue mosquito vector breeding through source reduction and larval control;

c. improving health education on dengue prevention.

(3) Reduce the incidence of diarrheal illness in school children.

(4) Reduce the number of absence episodes and length of those due to these illnesses.

## Methods/design

### Trial design

This is a 2×2 factorial cluster randomized controlled trial to study the effect of a set of diarrhea interventions (DIA) and a set of dengue interventions (DEN) delivered in rural primary schools in Colombia to eligible school children to reduce diarrheal disease and exposure to dengue vectors. Each school (cluster) is randomized to one of four study arms: DIA, DEN, DIADEN, and control (C). Randomization of study arms is stratified by municipality (two municipalities, that is, two levels). Control schools will carry out their normal activities without any intervention through this or any other project. A cluster design was considered the only feasible option for two main reasons. First, it would not be possible to evaluate the effect of the two individual interventions if they were both implemented in all the same schools. Second, it will be possible to evaluate the interventions as they would have been implemented in a practical disease control initiative. Furthermore, a factorial design allows comparison of separate and combined intervention sets with controls.

### Location and recruitment of participants

**Figure 1:**
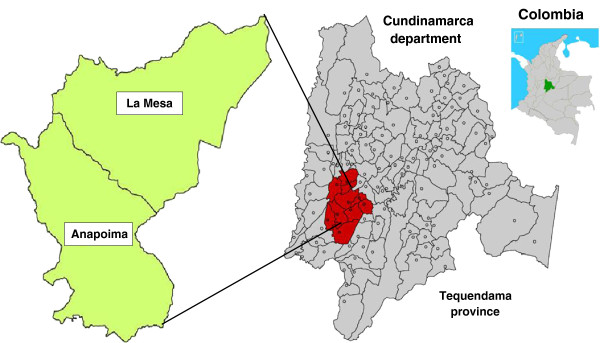
**Study sites.** Location of the two study municipalities La Mesa and Anapoima in Tequendama province, Cundinamarca department, Colombia.

The trial is carried out in rural primary schools in two municipalities, La Mesa and Anapoima, in Tequendama province, Cundinamarca department, Colombia (Figure 1). These municipalities were selected based on presence of diarrheal diseases and dengue in this province. Furthermore, the University El Bosque has as a policy to focus research in the Apulo river basin, where the two municipalities are located. We have conducted previous studies in the area related to environmental conditions in rural schools, water filters, solid waste, and dengue [33-35]. The reason for focusing on rural areas was due to the general lack of improved sanitation and access to improved sources of drinking water in rural areas [4] and reports of increasing risk of dengue transmission in rural areas [12,36]. The emphasis of schools in this study was because of the fact that school children are a natural entry point for health education activities aimed at improved community public health [37].

**Figure 2:**
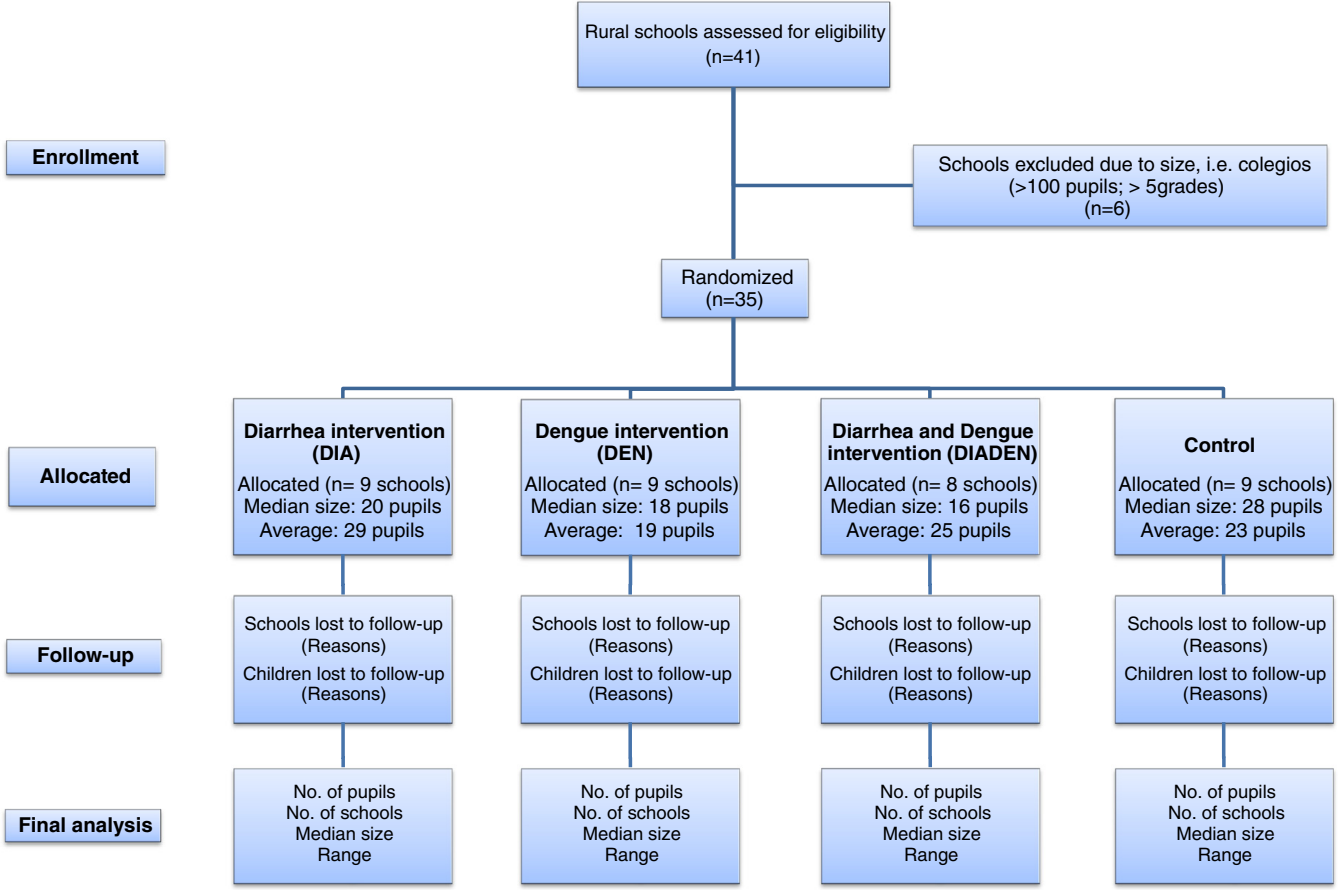
**Flow chart of school and pupil selection.** Selection of rural primary schools in La Mesa and Anapoima municipalities in Cundinamarca department, Colombia. Of the 35 schools randomized, one did not receive interventions because it was closed by landslides (therefore nine schools were allocated to DIA, but only n=8 will receive this intervention).

A list of all schools in the two municipalities was provided by the municipal administration in each municipality. Rural schools with pupils in grades 0 to 5 (ages 5 to 16 years) were selected from this list. The schools’ principals and teachers of each class with pupils in this age group were contacted by project staff, informed about the study, and invited to participate. A flow chart of school and pupil selection is shown in Figure 2.

#### School inclusion criteria

All rural primary schools, with grades 0 to 5, within the boundaries of the two municipalities with a written consent of the school teachers to participate will be included in the trial. Inclusion schools will not be involved in any diarrheal or dengue control program.

#### School exclusion criteria

Schools whose participation is considered infeasible will be excluded, for example large rural schools (*colegios*, with >100 pupils and > five grades), and those which are inaccessible or closed. Schools not wishing to participate in the trial will also be excluded.

#### Pupil inclusion criteria

All pupils in the participating rural schools will be recruited for follow-up. Both assent from the pupil, and written consent from a parent or guardian is required for children to participate in the trial. Newly enrolled children will also be eligible to participate, maintaining an open cohort.

#### Pupil exclusion criteria

Pupils without either assent or parental consent will not participate in the trial. Pupils will leave the study if they change enrolment to a school outside the study area.

### Interventions

Each set of interventions, DIA and DEN, targets various determinants or components of each disease as shown in Table 1.

**Table 1:** Diarrhea and dengue interventions implemented in rural primary schools in La Mesa and Anapoima municipalities, Cundinamarca, Colombia

**Component**	**Intervention**	**Frequency of intervention**	**Objectively verifiable indicators**	**Sources of verification**	**Expected outcome**
** (a) Diarrhea interventions (DIA) **					
**Drinking water quality**	Drinking water filters	Continuous	Values of water quality parameters^a^	Field collection	Clean water supply
	Cover drinking containers with lids	Continuous	Observational index: lid fitted correctly (yes/no)	Field observation	Clean water supply. Ensuring no additional contamination to water
	Cleaning water storage containers	At least once per semester	Observational index: appearance clean (yes/no)	Field observation + responsible municipal authority	Clean water supply. Ensuring no additional contamination to water
**Hygiene**	Promotion of hand-washing with soap	Daily	1. Presence of soap (yes/no).	Field observation survey (for indicator 1 and 2). Questionnaire (for indicator 3).	Hand-washing practices carried out and maintained (as taught in educational campaign)
			2. Availability of water for hand-washing (yes/no).		
			3. Frequency of hand-washing with soap by school pupils		
	Promotion of proper use and cleaning of toilets	Daily	Toilet cleanliness score	Field observation	Eliminate potential routes for feces ingestion during toilet use
**Education and training**	Educational campaign on diarrheal disease, hand-washing, hygiene, health and water relationships	Monthly modules	KAP score	KAP questionnaire	Children acquired proper hygiene and sanitation knowledge and practices
** (b) Dengue interventions (DEN) **					
**Adult mosquitoes**	Insecticide treated curtains	Continuous	*Aedes aegypti* adult mosquito density	Field collections	Reduce adult mosquito density
**Immature mosquitoes**	Cover containers with lids or covers	Continuous	*Aedes aegypti* larval and pupal density	Field collections	Reduce immature mosquito density
	Treatment with pyriproxifen in containers that cannot be fitted with lids or covers	Continuous with weekly follow-up	*Aedes aegypti* larval and pupal density	Field collections	Reduce immature mosquito density
**Solid waste management**	Larval source control through solid waste management	At least once per semester	Number of positive *Aedes aegypti* immature breeding sites in solid waste	Field observation	Elimination of breeding sites
**Education and training**	Educational campaign on dengue disease; vector biology, ecology, and control; role of solid waste; water and health relationships	Monthly modules	KAP score	KAP questionnaire	Children acquired knowledge and practices on dengue and mosquito control

^a^*In-situ*: temperature, pH, electrical conductivity, total dissolved solids; Laboratory: Total coliforms, fecal coliforms, and E. coli.

#### Diarrhea

Water filters will be installed to improve the quality of the drinking water used in schools. The ceramic filters, originally of pre-Columbian design, have been improved and promoted by the non-governmental organization Potters for Peace [38]. In Colombia the production and distribution of these filters are supported by the non-governmental organization Oxfam. All drinking water containers will be provided with fitted lids or nets covering the opening of the container. These containers will be cleaned once per semester by technical staff of each municipality. Teachers and children will oversee that containers remain covered. Interventions related to hygiene practices are the promotion of hand washing with soap and the proper use and cleaning of toilets. Soap will be provided by the project and pupils will be instructed how to wash their hands. Hand-washing will be carried out before eating and after toilet visits. Cleaning of toilets will be carried out on a daily basis by each school. The DIA educational campaign consists of project-designed educational and training guides on diarrheal disease, hand-washing, hygiene, and health and water relationships adjusted to the curricula of the children’s ages.

#### Dengue

To reduce dengue entomological risk factors, we will install insecticide-treated curtains to reduce mosquito entry into schools. Windows in all classrooms and computer rooms will be fitted with deltamethrin-treated curtains made from LifeNet® material produced by Bayer CropScience. LifeNet® is a long-lasting insecticide-treated material with deltamethrin incorporated into polypropylene fibers. LifeNet® has interim approval by the World Health Organization Pesticide Evaluation Scheme (WHOPES) for use in vector-borne disease control [39]. Insecticide susceptible tests were carried out on mosquitoes from the study area which revealed that mosquitoes were 100% susceptible to deltamethrin. In contact with the skin, the main adverse effect of pyrethroids such as deltamethrin may produce tingling and other sensations known as paresthesia [40]. A risk assessment of deltamethrin-treated bednets for malaria concluded that the ‘risk:benefit ratio is very favorable’ [41]. The study will not hold any liquid insecticides.

All water containers will be provided with fitted lids or nets covering the opening of the container that completely prevent the entry of mosquitoes. Teachers and children will regularly oversee that containers are covered/sealed. Those containers that cannot be fitted with lids or nets will be treated with pyriproxyfen (Sumilarv®, Sumimoto Chemical Company), an insect growth regulator which prevents the emergence of adult mosquitoes that is also approved by WHOPES [42]. Pyriproxyfen will be administered according to the producer’s guidelines. Larval source management will also be carried out by solid waste clean-up and collection campaigns arranged by teachers and project staff and carried out by pupils. These campaigns will be carried out at the beginning of each semester and when pupils return from holidays. The waste will be separated into biodegradable and non-biodegradable waste. The biodegradable waste will be managed in the school and the non-biodegradable waste will be stored safely until collected by the municipality administration once per semester. The DEN educational campaign consists of project-designed educational and training guides on dengue disease, vector biology/ecology/control, the role of solid waste as mosquito breeding sites; and water and health relationships with age-adjusted curricula.

Interventions will be implemented at the start of the school year (February to April 2012) and will continue for at least three complete school semesters (1.5 years) (four complete school semesters or 2 years may be possible, depending on funding). Supervision and management of interventions and adherence to the activities will be monitored monthly by teachers and the project team.

### Outcomes

The primary outcome measure for diarrhea will be the incidence rate of diarrhea in school children detected by school absence registers and parental confirmation. The primary outcome measure for dengue entomological risk will be *Ae. aegypti* adult density per school (Table 2).

**Table 2:** Primary and secondary outcome measures for evaluating diarrhea and dengue interventions in rural primary schools in La Mesa and Anapoima municipalities, Cundinamarca, Colombia

**Outcome**	**Collected by**	**Frequency of collection**	**Source**
***Primary outcomes***			
Incidence rate of diarrhea in school children	School absence registers and parental confirmation (telephone interview)	Recorded daily, collected weekly	Teachers; children’s parents
Density of adult female *Aedes aegypti* in each school (that is, number of mosquitoes collected per time unit)	Electric Prokopack aspirator in 10 to 15 min per classroom	At 4, 9, and 15 months post-intervention	Collections in schools
***Secondary outcomes***			
Breteau index (number of containers with *Ae. aegypti* immatures/100 schools)	Dippers and nets	At 4, 9, and 15 months post-intervention	Containers in schools
Number of pupil absence episodes and absence days due to diarrhea	School absence registers and parental confirmation (telephone interview)	Recorded daily, collected weekly	Teachers and parents of children
Number of pupil absence episodes and days due to probable dengue	School absence registers, parental confirmation (telephone interview), and health clinic confirmation. Probable cases defined based on WHO criteria [13]	Recorded daily, collected weekly	Teachers, parents, and health clinics
Number of pupil absence episodes and days due to all-cause illness	School absence registers and parental confirmation (telephone interview)	Recorded daily, collected weekly	Teachers and parents of children
Values of drinking water quality parameters^a^	Water samples	At 4, 9, and 15 months post-intervention	Drinking water containers in schools
Values of calculated KAP-scores	Questionnaires	At 4, 9, and 15 months post-intervention	School children

^a^*In-situ*: temperature, pH, electrical conductivity, total dissolved solids; Laboratory: Total coliforms, fecal coliforms, and E. coli.

The secondary outcome measures are the Breteau index (number of containers with *Ae. aegypti* immatures/100 schools) (other measures of immature dengue vector infestation will also be measured), number of pupil absence episodes and days absent due to diarrhea, number of pupil absence episodes and days absent due to probable dengue, number of pupil absence episodes and total days absent due to any other illnesses, a selected set of drinking water quality parameters, and values of calculated KAP scores (Table 2).

### Sample size

The sample size calculations were carried using a target number of participants of 873 pupils from 35 schools with an average of 25 pupils per cluster (school) (range, 5 to 96 pupils), and a harmonic mean of approximately 17. The latter was used in sample size calculations (see below) to allow for variation in school sizes.

For the diarrhea intervention, the sample size is in terms of numbers of schools and numbers of children per school. For the dengue intervention, the sample size is in terms of numbers of schools, since the primary endpoint will be measured only in schools. In other words, cluster-randomization applies to the diarrhea endpoint, but, in practice, not to the dengue endpoint, since there is only one school per cluster.

#### Diarrhea intervention

The sample size for the primary endpoint of diarrhea incidence was calculated using methods for cluster-randomized trials [43]. Existing data from the study area were used to estimate baseline diarrhea incidence (0.28/person-year) and within-school clustering (coefficient of variation *k* = 0.8). For 17 children per school followed up for 2 years, 35 schools achieve 90% power for a 75% reduction in incidence and 5% two-sided significance level.

#### Dengue intervention

Baseline values of the primary dengue endpoint (*Ae. aegypti* adult density in schools) were based on values reported from Mexican schools [44], as no comparable data were available from Colombia. To allow for over-dispersion relative to Poisson, a negative binomial distribution was fitted to these data, yielding a mean of 24 mosquitoes per schools and dispersion parameter (*θ*) of 0.75. Then, power was estimated using the method of Brooker *et al.*[45] which assumes equal numbers per arm. Taking the above parameters and 17 schools per arm, a 70% reduction in numbers is detectable with 84% power, and 75% reduction with 92% power. The power is slightly higher for 18 *vs.* 17 schools, which are the numbers randomized per arm for each of the two main comparisons (Figure 2).

### Sequence generation and allocation concealment

Anapoima and La Mesa municipalities differ in ways which are likely to be associated with the trial outcomes. For example, La Mesa schools are located at higher altitudes (712 to 1,610 meters above sea level) than Anapoima schools (588 to 1,089meters above sea level). Furthermore, only Anapoima has a municipal educational board which potentially improves educational follow-up. We therefore decided to stratify based on municipality [46]. Allocation of schools to the different trial arms was carried out at an open-to-the-public randomization event in each municipality before the start of the school year. After fully informing the school principals and teachers of the purpose of the event a raffle was arranged where a representative of each school drew a number indicating to which arm of the trial their schools would be allocated. To minimize bias at the time of data analysis each school was assigned a unique code to blind the statistician analyzing the data. All schools were thus allocated publicly at the start of the trial, obviating the need for allocation concealment. Control schools will receive the interventions at the end of the project if these interventions effectively affect the outcome measures.

### Statistical methods

For the diarrhea outcome, clustering will be taken into account by analyzing the school-level rates. The above sample size method [43] assumes the analysis will be done by a *t*-test comparing such summary rates between two arms. This is equivalent to analysis of covariance (ANCOVA) with a single binary explanatory variable. For the current trial, additional explanatory variables are required to allow for: (a) two interventions rather than one; and (b) the stratification by municipality. The response variable (school-level diarrhea incidence) may be log-transformed if this results in a more normal (Gaussian) distribution of the residuals. A secondary analysis will include a further binary explanatory variable representing the interaction term between the two interventions.

The dengue outcome, *Aedes aegypti* density, will be summed over follow-up time points to give a single rate per school. This will be analyzed by negative binomial regression using the number of adults, and the logarithm of the sampling effort (that is, person-time spent aspirating) as an offset. Hence, this analysis will yield density ratios. As for diarrhea incidence, the explanatory variables for the primary analysis will be trial arm and stratum.

Neither of the above primary analyses will include an interaction term although these will be estimated in secondary analyses.

### Data collection methods

Both quantitative and qualitative methods will be employed to address the objectives of this research. Data will be collected at 4, 9, and 15 months post-intervention, unless otherwise mentioned. The project will involve the following methods:

1. Health: Pupils’ absences from school for health reasons will be recorded and confirmed by phone calls to parents and, if necessary, house visits made by a health technician and supervised by the project physician. Probable dengue cases will be defined according to WHO criteria [13]. Data for pupil absences will be recorded daily by teachers and collected weekly by project staff.

2. Entomology: Dengue vector infestation and abundance will be evaluated by adult and immature collections. Adult mosquito collections will be carried out inside schools with a battery-driven Prokopack aspirator [47] for 10 to 15 min in each classroom. Immature collections will be carried out in containers in schools. The immature collection enables the calculation of various immature indices, of which the Breteau index (number of positive containers/100 schools) and the number of pupae in relation to human population (number of *Ae. aegypti* pupae/person) are the most important. *Aedes* pupal productivity survey methodology allows for identification of the most epidemiologically important container types [48]. Water-holding containers in schools and their peri-domestic environments will be identified, counted, measured, and classified according to shape (S), use (U), and material (M) using the standardized container classification methodology (SUM-method) [49]. Mosquitoes will be identified in the field laboratory using common identification keys (for example, [50,51]).

3. Water quality: Fecal contamination and physicochemical parameters of stored water in schools. Presence of *E. coli* will be used as a proxy for risk of diarrheal infections according to WHO guidelines [6]. In each selected school, samples from all drinking water containers and taps will be collected for both *in situ* tests (temperature, pH, electrical conductivity, and total dissolved solids) and laboratory analysis (fecal coliforms and *E. coli*) following standard methods [52]. Each sample bottle will be labeled with school and container codes and transported on ice to a certified laboratory (Daphnia Laboratory, Bogotá, Colombia) for analysis.

4. Knowledge, attitudes, and practices: Pre-designed questionnaires on the knowledge attitudes and practices will be administered to pupils and teachers.

5. Location: The geographical location of each school will be recorded by a handheld GPS and plotted in a GIS using available digitized base maps for spatial analyses and for presentation purposes.

6. Climate: General climate data (rainfall, temperature, and humidity) will be retrieved from hydrometereological stations of the Corporacion Autonoma Regional de Cundinamarca (CAR) for the Tequendama province, of which two are located in La Mesa municipality. At a local level, climate data will be collected in three selected schools in each municipality, based on differences in altitude and precipitation. Indoor maximum and minimum temperatures, indoor humidity, and rainfall will be measured twice daily (07:00 and 12:00) during school days. Data will be collected by school children with supervision of teachers.

### Publication policy

The principal investigators will ensure that the results of this trial are published regardless of outcomes. Reporting of the trial results will follow the guidelines of the CONSORT statement [32].

### Ethical review

The study will be conducted according to the Declaration of Helsinki and the International Guidelines for Ethical Review of Epidemiological Studies. This trial was approved by the Comité Institucional de Ética en Investigaciones de la Universidad El Bosque, Bogotá, Colombia on 30 August 2011 (Acta No. 146). The trial protocol was reviewed by the Regional Committees for Medical and Health Research Ethics (REC) in Norway.

## Discussion

To our knowledge this is the first trial investigating the effect of a set of interventions to control both diarrheal diseases and dengue fever. This is also the first trial to study the combination of diarrhea-dengue disease control in school settings. Diarrheal diseases originating from contaminated drinking water due to poor water collection and storage practices may be epidemiologically linked to dengue fever, whose mosquito vectors breed in stored water containers. Thus, containers used to store water may be the link between the two diseases. By integrating disease interventions it may be possible to effectively and cost-efficiently control disease outcomes. A 2×2 factorial design is a logical choice for evaluating two related sets of interventions.

The study area is fairly suitable for the current study as both diseases are prevalent in the two selected municipalities. We are also familiar with the study area from previous studies conducted there. It was decided to set the trial in small rural primary schools for several reasons: rural areas are often neglected in terms of national health policies which is reflected in reports on global rural–urban disparities for access to safe water and improved sanitation [4]; dengue control primarily takes place in urban areas, although dengue transmission is also a problem in rural areas [12,36]; small rural schools are logistically feasible and fairly uncomplicated to manage (although sometimes difficult to access during the rainy season). In Colombia between 13% and 29% of dengue cases are reported from rural areas [53-55], with the highest number from the present study area [55]. Finally, only relatively few studies have investigated the effect of health interventions in schools in developing countries (for example, [56,57]). Further, school children may act as mediators of health messages to their parents and the community [58]. With the interventions being implemented at the school level, it follows that the school should also be the unit of randomization, resulting in a cluster-randomized design. Our primary diarrhea endpoint is disease, but this is not the case for dengue. For diarrhea, parents are best placed to evaluate the WHO definition of diarrhea in terms of stool frequency and consistency, while dengue cases are difficult and expensive to confirm by laboratory tests and statistical power is problematic due to large between-year variation in incidence. Hence we have chosen a dengue vector endpoint, specifically the density of adult *Aedes* mosquitoes, this being more proximally related to disease than the *Stegomyia* indices used in some previous trials.

It should be noted that the current study design evaluates two sets of interventions and so, within each set, the effects of single interventions on the outcome measures cannot be distinguished. It is the overall effect of each of these two sets of disease-specific interventions that is of interest in this study.

### Trial status

At the time of submission of this manuscript (June 2012) the trial had completed the baseline data collections, enrollment of schools, and randomized allocation of schools to the four study arms (Figure 2).

## Competing interests

The authors declare that they have no competing interests.

## Authors’ contributions

HJO and TAS, principal investigators, conceived and secured support for this project. All authors were involved in trial design. HJO wrote the first draft of this manuscript. NA wrote the sections on sample size calculations. All authors contributed to revisions of the manuscript, have read and approved the final version for publication, and take public responsibility for its content.
